# TARGETED TOOL TO MEASURE DEMENTIA LITERACY IN PREVENTION AND TREATMENT AMONG SEXUAL AND GENDER MINORITY OLDER ADULTS

**DOI:** 10.1093/geroni/igz038.444

**Published:** 2019-11-08

**Authors:** Maritza Dowling, Dana Hines

**Affiliations:** 1 George Washington University, Washington, District of Columbia, United States; 2 George Washington University, Washington, United States

## Abstract

Dementia prevalence among sexual minority adults age 60+ in the US is approximately 7.4%. This represents over 200,000 sexual minority older adults living with Alzheimer’s disease (AD). Compared to the general aging population, LGBTQ older adults face unique challenges throughout their life-course that may increase exposure to risk factors associated with neurocognitive dysfunction and progression to dementia. Culturally-competent instruments to measure dementia literacy are needed to inform the design of targeted educational interventions for early diagnosis and improved health outcomes. We sought to develop and test a measure of dementia literacy in a community sample of LBGTQ older adults. Following a comprehensive analysis of existing scales, items were adapted resulting in a 35-item survey containing subjective and objective literacy measures. Participants were recruited through community centers in Washington, DC and Maryland. Forty LGBTQ older adults (50-80 years) completed the questionnaire. The analysis of these data was used to modify the original survey. A final 25-item questionnaire was administered to another sample of 50 individuals. 60% correctly identified dementia symptoms from vignettes. Nearly-half believed that dementia symptoms could be reduced by lifestyle changes and drug therapies. Commonly identified risks for dementia included aging, family history, genetics, stroke, and brain injuries. Frequently-endorsed methods for risk reduction included physical activity, intellectual/brain training and social activities. Education level, age, and socioeconomic variables were strongly associated with literacy. Most respondents did not associate cardiovascular/metabolic risk factors with dementia risk. Knowledge of risk factors underlying AD may help underserved populations close current health disparities gaps.

